# Supplementary material for: Co-Creation of Massive Open Online Courses to Improve Digital Health Literacy in Pregnant and Lactating Women
Source: Int J Environ Res Public Health. 2022 Jan 14;19(2):913. doi: 10.3390/ijerph19020913 (PMC8775560; doi:10.3390/ijerph19020913)

S1 Image. Screenshot of the platform for co-creation activities

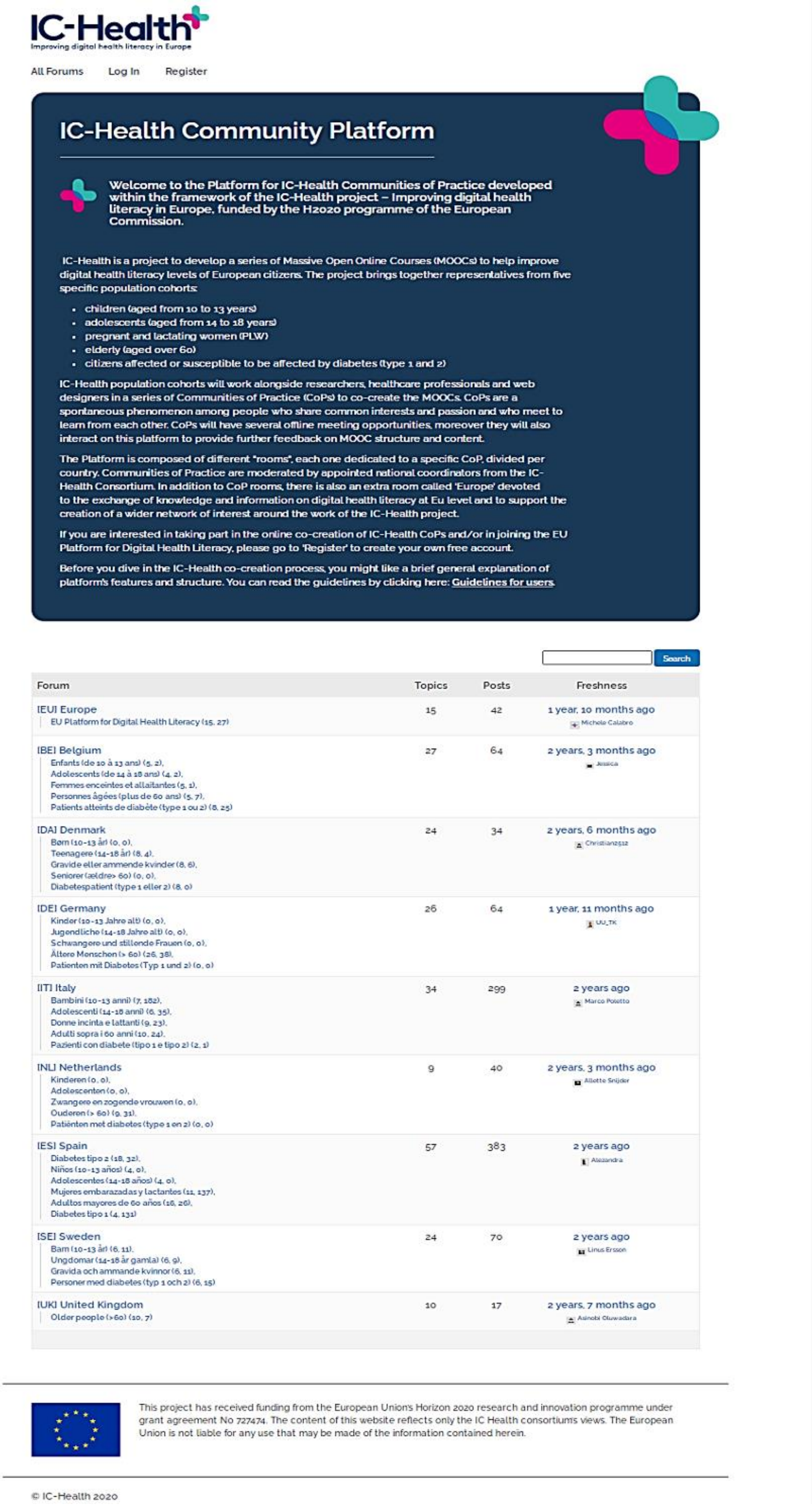

S2 Table. Illustrative quotes from Focus Groups

Experience/general opinion using internet for health and illness issues

All mothers agreed that internet has never or hardly ever been the only or first source of information they consulted to get health-related information during pregnancy or after delivery. In most cases, they used internet because of failure in the communication with health professionals.

- *"When doctors are not available or when the human touch is missing, internet can compensate this lack of empathy by health personnel".*
- *"Doctors quite often have different opinions about health issues. How to treat disease, how to behave, etc. So, internet can help to compare these views and take a decision".*
- *"If doctors talk too technical, I don't understand".*
- *"When I was seven-months pregnant I went the Emergency Room very upset because I had some contractions, and the midwifes really treated me badly, screaming at me… So, which is patients' reaction in these cases? They go home and the next time, rather than going back to the hospital, look on the web".*

In the case of lactating women, they consider it can be beneficial when it is difficult to move from home, for example in the postpartum period when it is difficult to move around with a small baby. The most relevant benefit is the chance to find information and knowledge which can empower the person and know what is available and have things done the way one prefers. In fact, some say they have made changes thanks to the information they found on the Internet.

- *"Empower yourself. Because the only way to change things is that patients get empowered and that when you reach there, they cannot do whatever they want, and this we have to change it".*

Finding other people experiences is another of the benefits participants recall, it is a relief if you find other people who have something like you.

Participants' needs and expectations of the use of Internet as source of information on health and illness issues

Participants expressed different and varied informational needs, including:

- Information on pregnancy in general (e.g., phases) and more specific information about the aim and type of periodical texts/exams pregnant mothers are due to throughout pregnancy
- Information on delivery, to get prepared to it
- Information on how to donate milk or umbilical cord after delivery
- Information about the possibility/process/opportunities of freezing of stem cells after delivery
- Vaccine/vaccination
- Drugs leaflets and side-effects
- Availability of alternative care
- Information on weaning and feeding induction, with focus on self-managed weaning
- Information on diseases/illnesses/conditions they had never heard about before or they are not familiar with

Participants' trust on internet as source of information on health and illness issues

Most participants could not clearly identify situations/topics for which they would not trust internet as source of information. Only one mother clearly stated she never consulted internet (and will ever do it) about vaccines/vaccination.

- *"I refused to look on the internet about vaccines. I know the web is full of fake news about it and I simply didn't want to assist or take part to that debate".*

Another mother stressed on the poor utility of internet when the required information is more 'emotional' and entails a great deal of empathy (e.g., information on bodily changes during pregnancy).

- *"Issues like ante-natal courses and preparation to delivery cannot be fully understood if read... you need sharing views with other peers, to listen to midwives' indications, to make questions… maybe a webinar can help, but certainly websites are not enough".*

Most mothers considered information on health/illness found on the internet trustful when:

- The source of information is known and authoritative (e.g., scientific sources or health professionals' opinion)
- Multiple sites confirm/report it
- The information found on internet was shared, discussed and confirmed also by experts / health professionals

- *"I trusted that website since information was provided by a registered and referenced midwife, not anonymous, with full credentials disclosed".*

- *"I tend to verify the source of information… I look for sites linked to health institutions, health professionals' orders, research centres. I don't link not science-based sites".*
- *"When I look for information on the internet, I always try to compare information retrieved from different sites and I check the nature of the website... for example when the site is sponsored by a commercial brand, I am more cautions than when it is linked to scientific bodies".*

Perception of the use of internet as source of information on health and illness issues by other people
In general, participants would not recommend to their peers the use of internet as source for information on health, due to the combination of widespread poor-quality information (e.g., on vaccines and vaccination) and most people's lack of capacities to properly distinguish between reliable and not reliable sources. In case internet is used, they would recommend paying attention to the source of information (e.g., sites which may aim is to promote/sell products are not recommended) and to compare different sources, paying attention to their authority.<br>Also, many participants would encourage medical doctors to consider internet not only as a threat to their authority but as a possible ally in informing or educating patients/mothers, by recommending the consultation of reliable websites.<br><br>– *"Once I spoke with my gyn about googoling health information, she told doctors are fully aware of this practice among patients and think this habit affects their work".*

S3 Image. Screenshot of general appearance of the Spanish MOOCs updated version

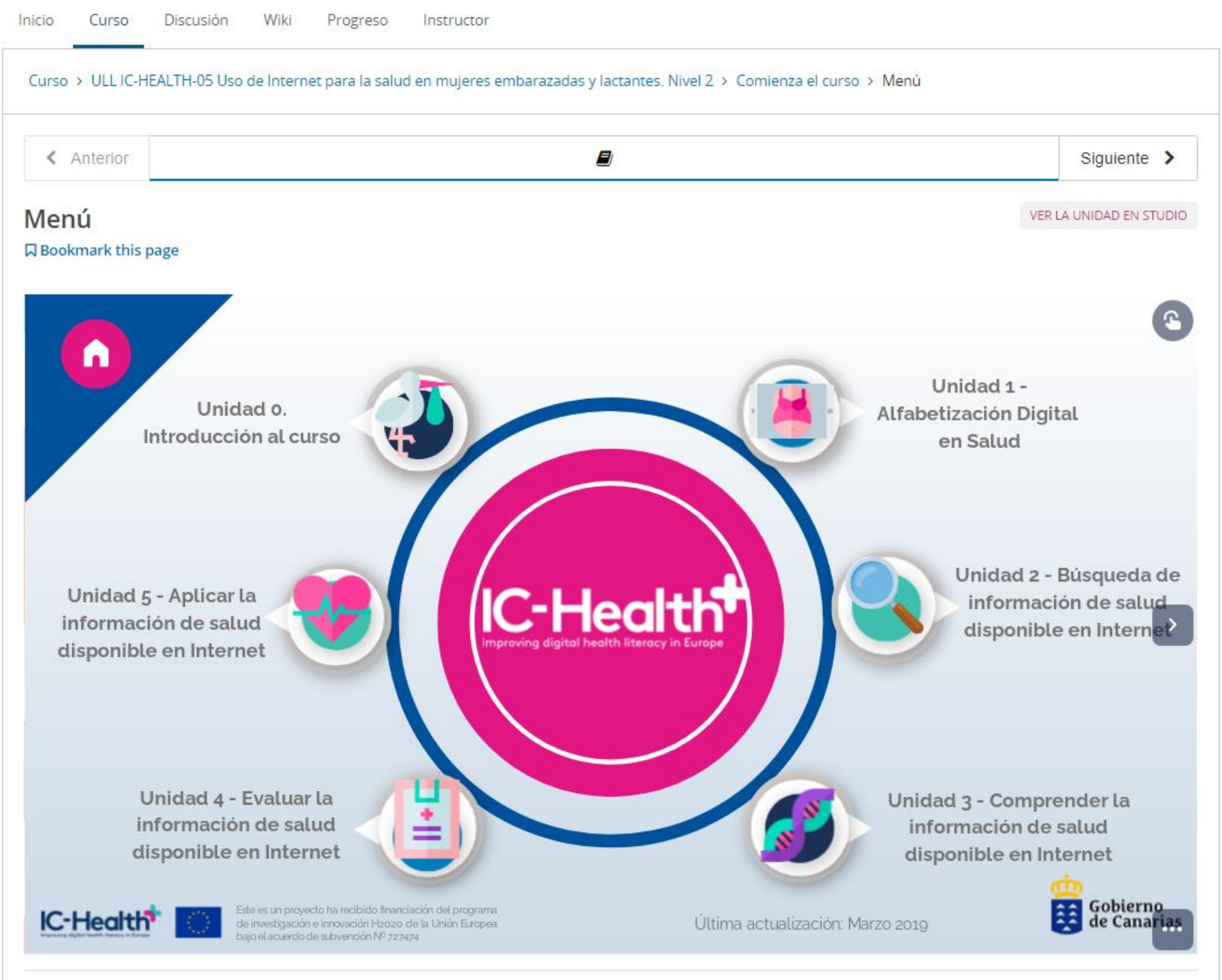

S4 Image. Screenshot of an example of the content of the unit "Find" of the Spanish MOOCs updated version

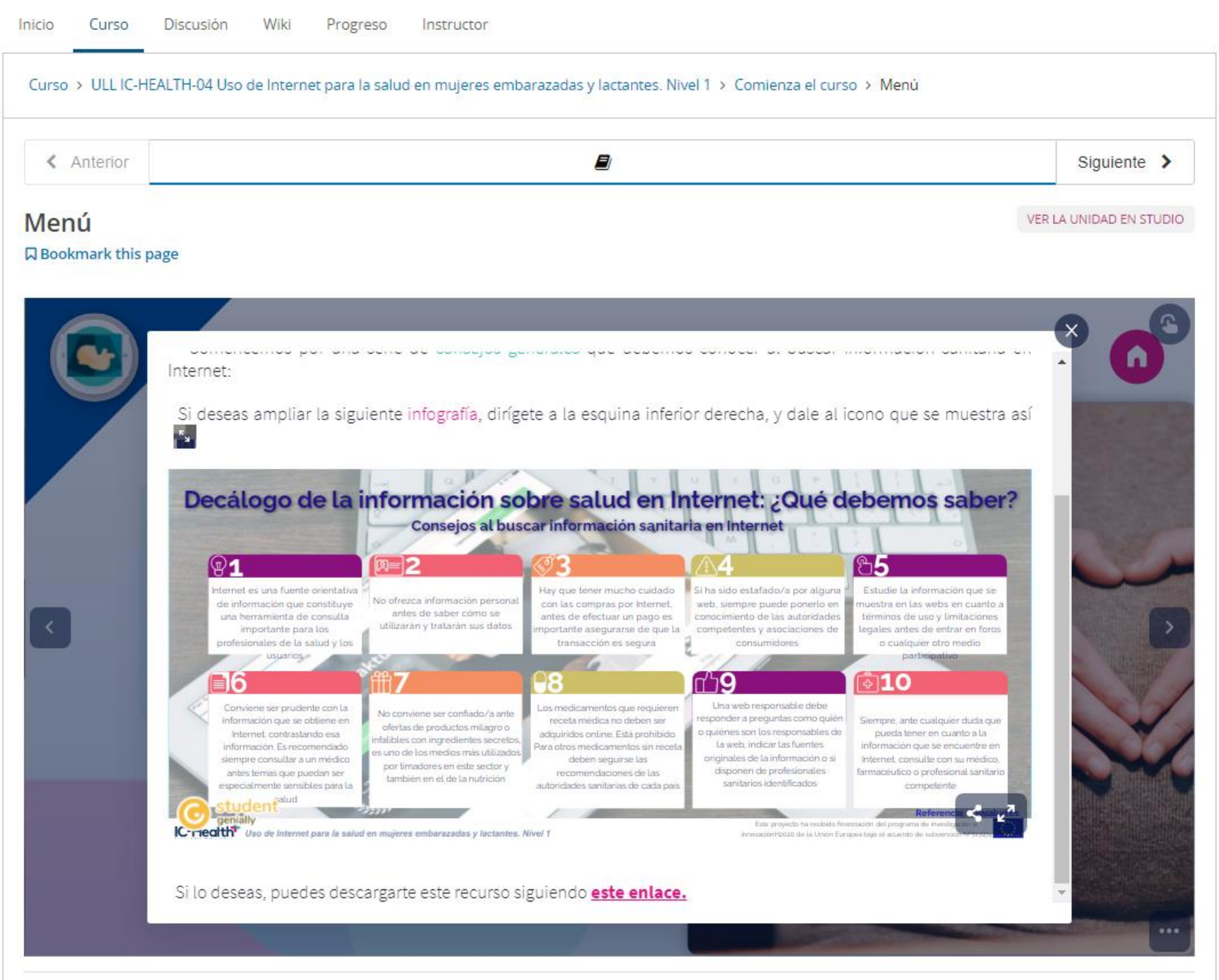

Supplement: Supplementary file 1 [file ijerph-19-00913-s001.zip › ijerph-1484204-supplementary/ijerph-1484204-supplementary.pdf]
