# Supplementary material for: Co-Creation of Massive Open Online Courses to Improve Digital Health Literacy in Pregnant and Lactating Women
Source: Int J Environ Res Public Health. 2022 Jan 14;19(2):913. doi: 10.3390/ijerph19020913 (PMC8775560; doi:10.3390/ijerph19020913)
Supplement: Supplementary file 1 [file ijerph-19-00913-s001.zip › ijerph-1484204-supplementary/supplementary.pdf]

Table S5. Membership of the IC-Health project partner consortium.

Canary Islands Government—General Directorate for Public Health	Spain
Associazione Comitato Collaborazione Medica	Italy
University of La Laguna	Spain
Université Catholique de Louvain	Belgium
University of Udine	Italy
Ulster University	UK
Tallinn University	Estonia
University of Las Palmas de Gran Canaria	Spain
Consiglio Nazionale delle Ricerche	Italy
Scanbalt	Denmark
Meyer Children's Hospital	Italy
Consulta Europa Projects & Innovation	Spain
FUNKA NU AB	Sweden
European Health Management Association	Belgium
